# The Draft Genome of *Deladenus siricidicola*

**DOI:** 10.21307/jofnem-2019-036

**Published:** 2019-07-29

**Authors:** Alisa Postma, X. Osmond Mlonyeni, Frederick Clasen, Fourie Joubert, Bernard Slippers

**Affiliations:** 1 Department of Biochemistry, Genetics and Microbiology, Forestry and Agricultural Biotechnology Institute, University of Pretoria, Pretoria, South Africa; 2 Department of Biochemistry, Genetics and Microbiology, Centre for Bioinformatics and Computational Biology, University of Pretoria, Pretoria, South Africa; 3 Cancer Metabolism Laboratory, The Francis Crick Institute, London, UK; 4 Centre for Host-Microbiome Interactions, Dental Institute, King’s College London, London, UK

**Keywords:** Biological control, *Deladenus siricidicola*, Entomoparasitic nematode, Genome, Sirex noctilio

## Abstract

The nematode *Deladenus siricidicola* is used as biological control agent against the invasive woodwasp *Sirex noctilio*, a serious invasive pest of *Pinus* plantations globally. The draft genome of this ecologically and economically important entomoparasitic nematode was determined.

The number of invasive insect pests affecting forestry and agriculture globally is rapidly increasing (Ramsfield et al., 2016). The Sirex woodwasp, *Sirex noctilio* (Hymenoptera: Siricidae), along with its symbiotic fungus, *Amylostereum areolatum* (Russulales: Amylostereaceae), are amongst the most important invasive pests of *Pinus* trees globally and has caused billions of dollars’ worth of losses since its first detection in New Zealand in the early 1900s (Slippers et al., 2012). The most effective control of *S. noctilio* is via the biological control agent, *Deladenus siricidicola* (Tylenchida: Neotylenchidae). This nematode has a bicyclic life-cycle, where in the free-living phase it reproduces in wood whilst feeding on *A. areolatum*, while in the parasitic phase it infects *S. noctilio* larvae and results in sterilized females. The parasitized adult female *S. noctilio* becomes the natural vector that disperses *D. siricidicola* into new trees.

In this study, the genome of *D. siricidicola* was sequenced and assembled. These data will be useful to understand the evolution and biology underlying the unique symbiotic relationships within this multipartite symbiotic system, including the genomic features linked to the adaptation to the bicyclic lifestyle of this nematode.

A *D. siricidicola* strain known as the “Kamona strain,” that is widely used in biological control of *S. noctilio* in the Southern Hemisphere, was used for genomic DNA extraction using the phenol-chloroform method (Mlonyeni et al., 2011). Two single-end shotgun libraries were sequenced using Roche 454 technology at Inqaba, South Africa. Three paired end and two mate pair (~2 and ~5 kb inserts) libraries were sequenced using Illumina HiSeq and MiSeq platforms at Inqaba, South Africa and Fasteris SA, Switzerland. All DNA samples qualified based on quality criteria set by the sequencing facilities, with DNA concentrations measured above 4 μg and sample volumes above 50 μl (Table A1). The data obtained produced a genome coverage of ~90× the estimated genome size.

The quality of all raw data was analysed using FastQC (www.bioinformatics.bbsrc.ac.uk/projects/fastqc) and quality trimming and filtering were performed on Illumina data using Trimmomatic (Bolger et al., 2014) with customised parameters relevant for each library (Table A2). Roche 454 reads were assembled using Newbler/GS De Novo Assembler with default parameters and the resulting long contigs were incorporated into a first draft assembly together with the Illumina paired-end reads using VelvetOptimiser with a kmer-size of 77 and a coverage cutoff of 2× (Zerbino and Birney, 2008). The resulting paired-end assembly was scaffolded with available mate pair data using SSPACE (Boetzer et al., 2011). This produced a draft assembly ~100.56 Mb in size, containing 3,169 contigs with an N50 of 124 kb. The CEGMA program (Parra et al., 2007) was used to confirm genome completeness by searching for the presence of 248 core eukaryotic genes. The completeness of the *D. siricidicola* genome assembly was estimated at 92%. The assembly and genome completeness statistics are comparable to other nematodes in the Tylenchida order, such as *Meloidogyne incognita* (size: 122 Mb, 83% complete) and *Meloidogyne arenaria* (size: 163 Mb, 91% complete) (Szitenberg et al., 2017).

These data provide a valuable resource to study the evolution and biology of this nematode, specifically considering the adaptations necessary to sustain its unique symbiotic relationship with *S. noctilio* and *A. areolatum*.

GenBank accession numbers: the raw DNA sequence data and genome assembly were deposited at GenBank under BioSample No. SAMN10502236.

## Appendix

**Table A1.** DNA samples and concentrations.

Sample name	Concentration (ng/µl)
Arg1	3,486
Arg3	2,544
Arg4	2,337
Arg7	2,650
Ds7	990
Ds8	416.2

**Table A2.** Illumina library information.

Sequencing type	Library name/files	Library type	Read length	Number of reads	GC content (%)	Approximate coverage of genome	Trimmomatic parameters*
Illumina HiSeq	121119_SN1126_M_L001_GSA-35_R1.NoAdaptPR.fastq	Paired end	250 bp	13,126,801	35	27×	HEADCROP: 10 CROP: 240 LEADING: 30 TRAILING: 30 MINLEN: 200
	121119_SN1126_M_L001_GSA-35_R2.NoAdaptPR.fastq	Paired end	250 bp	13,126,801	35	27×	HEADCROP: 10 CROP: 240 LEADING: 30 TRAILING: 30 MINLEN: 200
Illumina MiSeq	AlisaPostma_S2_L001_R1_001.fastq	Paired end	250 bp	3,845,668	41	8×	HEADCROP: 10 CROP: 160 LEADING: 30 TRAILING: 30 MINLEN: 100
	AlisaPostma_S2_L001_R2_001.fastq	Paired end	250 bp	3,845,668	41	8×	HEADCROP: 10 CROP: 160 LEADING: 30 TRAILING: 30 MINLEN: 100
	Deladenus_S1_L001_R1_001.fastq	Paired end	250 bp	4,996,896	38	10×	HEADCROP: 5 CROP: 230 LEADING: 30 TRAILING: 30 MINLEN: 200
	Deladenus_S1_L001_R2_001.fastq	Paired end	250 bp	4,996,896	39	10×	HEADCROP: 5 CROP: 230 LEADING: 30 TRAILING: 30 MINLEN: 200
Illumina HiSeq	140528_SND104_B_L008_GSA-39_R1.RD30.NotEmpty.LinkerTrimmed-50bp-PR.fastq	Mate pair	50 bp	4,076,324	39	3×	HEADCROP: 5 LEADING: 30 TRAILING: 30 MINLEN: 35
	140528_SND104_B_L008_GSA-39_R2.RD30.NotEmpty.LinkerTrimmed-50bp-PR.fastq	Mate pair	50 bp	4,076,324	39		HEADCROP: 5 LEADING: 30 TRAILING: 30 MINLEN: 35
	140528_SND104_B_L008_GSA-40_R1.RD30.NotEmpty.LinkerTrimmed-50bp-PR.fastq	Mate pair	50 bp	9,097,316	37	7×	HEADCROP: 5 LEADING: 30 TRAILING: 30 MINLEN: 35
	140528_SND104_B_L008_GSA-40_R2.RD30.NotEmpty.LinkerTrimmed-50bp-PR.fastq	Mate pair	50 bp	9,097,316	37		HEADCROP: 5 LEADING: 30 TRAILING: 30 MINLEN: 35

Notes: *All Illumina libraries were subjected to the same Illuminaclip parameters: using a specified adapter file, trimmomatic searched for seed matches allowing maximally two mismatches. These seeds were extended and clipped if a paired read scored 30 or a single read scored 10.
